# LC-MS/MS analysis of the dog serum phosphoproteome reveals novel and conserved phosphorylation sites: Phosphoprotein patterns in babesiosis caused by *Babesia canis*, a case study

**DOI:** 10.1371/journal.pone.0207245

**Published:** 2018-11-28

**Authors:** Asier Galán, Anita Horvatić, Josipa Kuleš, Petra Bilić, Jelena Gotić, Vladimir Mrljak

**Affiliations:** 1 ERA Chair”VetMedZg”, Clinic for Internal diseases, Faculty of Veterinary Medicine, University of Zagreb, Heinzelova 55, Zagreb, Croatia; 2 Clinic for Internal Diseases, Faculty of Veterinary Medicine, University of Zagreb, Heinzelova 55, Zagreb, Croatia; Universita degli Studi di Padova, ITALY

## Abstract

Phosphorylation is the most commonly studied protein post-translational modification (PTM) in biological systems due to its importance in controlling cell division, survival, growth, etc. Despite the thorough research in phosphoproteomics of cells and tissues there is little information on circulating phosphoproteins. We compared serum from 10 healthy dogs and 10 dogs affected by *B*. *canis*-caused babesiosis with no organ dysfunctions by employing gel-free LC-MS/MS analysis of individual samples and tandem mass tag (TMT) label-based quantitative analyses of pools, both supported by phosphopeptide enrichment. Results showed a moderate number of phosphorylated proteins (50–55), with 89 phosphorylation sites not previously published for dogs although a number of them matched phosphorylation sites found in mammalian orthologs. Three phosphopeptides showed significant variation in babesiosis-affected dog sera compared to controls: Serum amyloid A (SAA) phosphorylated at serine 101 (up-regulation), kininogen 1 phosphorylated at threonine 326, and fibrinogen α phosphorylated at both threonine 20 and serine 22 (down-regulation). 71.9% of the detected phosphorylated sites were phosphoserine, 16.8% phosphothreonine and only 11.2% phosphotyrosine residues. TMT label-based quantitative analysis showed α-2-HS-glycoprotein / Fetuin A to be the most abundant phosphoprotein (50–70% of all phosphoproteins) followed by kininogen-1 (10–20%). The alterations of phosphorylated proteins observed in canine babesiosis caused by *Babesia canis* suggest new insights into the largely neglected role of extracellular protein phosphorylation in health and disease, encouraging urgent further research on this area. To the best of our knowledge the present study represents the first attempt to characterize canine serum phosphoproteome.

## Introduction

Protein phosphorylation is the most widespread known post-translational modification involved in almost all cellular events, controlling the most diverse cellular pathways [1]. At least one third of human proteins are phosphorylated, frequently at multiple sites and transiently [2]. Phosphorylation is a dynamic and reversible process modulated by a battery of protein phosphatases catalyzing phosphorylation–dephosphorylation reactions. Metabolic changes observed in many diseases can occur through changes to the expression of metabolic enzymes, the concentrations of substrates or products of those enzymes or PTMs of the proteins that facilitate these reactions. However, in many cases the role of phosphorylation is not clearly understood, especially in the case of extracellular circulating proteins. This limitation, together with technical drawbacks common to phosphoproteomic analysis (associated with the lability of phosphate groups) has hampered the analysis of phosphorylation of the most frequently available clinical samples [3, 4] (blood, serum, plasma, etc.). Additionally, phosphorylation / dephosphorylation of extracellular domains of matrix, cell-surface and trans-membrane proteins by ecto-kinases and phosphatases is an important regulatory mechanism in health and disease [5–8]. Stimuli-dependent secretion of ATP-containing vesicles by exocytosis as a way to control extracellular protein phosphorylation is gaining much attention in the regulation of phosphorylation of extracellular proteins responsible for long-term cellular adaptation [6]. On the other hand, protein conformational changes induced by phosphorylation could play an important role in fine-tuning protein activity [9] and conformational stability [10].

The increase in sensitivity of high-throughput proteomic techniques has allowed the study of protein phosphorylation in an extensive way and favored the opening of new horizons in biomedical research. Limited biomarker discovery studies for differential protein levels has resulted in a disappointingly low rate of application of biomarkers in clinical practice [3]. A revision of this problem has led to the development of emerging branches in proteomics applied to biomarker discovery: phosphoproteomics and glycoproteomics. Large-scale analysis of complex phosphoproteomes is a highly challenging task, undertaken principally by qualitative and quantitative tandem mass spectrometry (MS/MS) during the last few years. MS/MS offers both high sensitivity and throughput, and it is capable of determining both protein identity and precise location of phosphorylation site(s) [1]. However, comprehensive analysis of protein phosphorylation by MS continues to show some limitations, such as the lability of phosphate moieties, which can be lost during sample preparation and MS analysis, and the scarcity of phosphorylated proteins [11]. Phosphoprotein databases are available for a number of microorganisms, plant and animal species [12], though some animal species such as dogs are under-represented. On the other hand, software for predicting phosphorylation sites in a given protein sequence, for example GPS 2.0 and NetPhos 2.0, yields a maximum of 83% fidelity [13].

Qualitative differences can be inferred from the presence or absence of detected sites calculated from the relative number of times a site is identified in different samples (spectral counting) but this method is considered rather semi-quantitative due to lack of accuracy [14]. Unlike label-free approaches, stable isotope labeling allows the relative or absolute quantification and multiplexed quantification of peptides/proteins. The most widespread labels are covalent tags, such as the isobaric tag for relative and absolute quantification (iTRAQ) or the Tandem Mass Tag (TMT) [15].

Along with MS/MS, the optimization of phosphoprotein/phosphopeptide enrichment procedures has greatly contributed to the expansion of phosphoproteomics [16]. Recent studies of phosphoproteins in biological fluids include serum and plasma [4, 17], cerebrospinal fluid [18], saliva [19] and urine [20]. Abnormal protein phosphorylation is associated with many important diseases. Namely, alteration of phosphoproteins in biofluids has been reported as potential biomarker for several cancer types (breast, in case of phosphoproteins in circulating extracellular vesicles [21], pancreas [22], prostate [23] hepatocellular [24], etc.), autoimmune diseases such as psoriasis [25] and genetic diseases such as Duchenne muscular dystrophy [26]_._ Furthermore, the phosphorylation state of abundant phosphorylated serum proteins has been used as a marker of aging [27], schizophrenia [28], etc.

Despite emerging interest, there is little knowledge about serum/plasma phosphoproteins as markers for infectious diseases. Some phospho-proteins/-peptides are components of the pathogens in the host and can be converted into circulating or membrane associated antigens [29]. On the other hand, pathogen–induced inflammation could trigger modifications in phosphorylation of acute phase-related proteins and other proteins, but despite known kinases are involved in phosphorylation of proteins secreted to circulation (with a remarkable predominance of acidophilic kinases [30]), and in most cases the function of phosphorylation is poorly characterized [31].

Canine babesiosis is a significant tick-borne disease caused by several intraerythrocytic protozoan species of the genus Babesia. This pathology is most frequently caused by *Babesia canis*, present in North America, Southern Europe, parts of Africa and Asia. Several markers have been identified to be useful for prognosis and for monitoring after antibabesial therapy [32).[33, 34]. Cytokines have also been suggested as markers associated to the occurrence of complications in canine babesiosis elicited by several babesial species [35, 36].

Interestingly, a polymorphic membrane phosphoprotein (BrEMA1) has been described on the plasma membrane of *Babesia Rossi*- infected erythrocytes that is suspected to constitute a major virulence factor [37]. *Babesia canis-*caused infection could potentially contribute with the parasite’s phosphoproteins to the canine serum proteome, acting as biomarkers of the infection. Some other phosphoproteins such as *Trypanosoma cruzi’s* calcineurin B, a phosphatase, are involved in host cell invasion [38] and ecto-kinases have been found to be secreted by *Leishmania major* [39]. Moreover, phosphorylation of extracellular proteins can operate in signaling pathways responsible for cellular adaptation [5, 6], making serum an untapped source of phosphoproteins suitable for better understanding disease pathophysiology and discovery of disease biomarkers [28].

The present study shows an approach to characterize qualitatively individual serum samples and quantitatively pools containing the same 10 control samples and 10 samples from dogs suffering from babesiosis before the onset of organic dysfunction [40]. In order to test alterations in serum phosphoproteins as potential markers for babesiosis, we have used state-of-the-art phosphopeptide enrichment procedures and optimized LC-MS/MS protocols to maximize the detection of phosphorylation sites. Combined qualitative and quantitative analyses offer novel data on the alteration of phosphoprotein levels in babesiosis, a hemolytic infection closely related to malaria and sepsis and the concomitant acute systemic inflammation. Variations observed could represent candidate biomarkers for the early detection of babesiosis in dogs and show potential usefulness in clinical applications.

## Materials and methods

### Animals

The study was performed on two groups of animals. Group 1 consisted of 10 dogs naturally infected by *Babesia canis (B*. *canis)*, admitted to the Clinic for Internal Diseases, Faculty of Veterinary Medicine, University of Zagreb, Croatia, with clinical signs of acute babesiosis. Dogs in this group were of various breeds, between 1 and 14 years of age and 6 of them were males. The clinical manifestations were variable and included anorexia, lethargy and fever, ticks found by the owner or veterinarian, pale mucous membranes, anaemia, jaundice, hemoglobinuria or hematuria, splenomegaly, tachycardia and vomiting. The main complication was the development of an excessive inflammatory response called “systemic inflammatory response syndrome” or SIRS^61^. The diagnosis was confirmed by demonstration of the parasites within infected erythrocytes in Romanowsky-stained thin blood smears. One dose (6 mg/kg of body weight) of imidocarb dipropionate (Imizol, Shering–Plough) was administered to all dogs subcutaneously on the day of admission. Additional treatment consisted of various fluids (colloid and crystalloid therapy), and whole blood transfusion when this was indicated. Subspecies were confirmed using PCR (polymerase chain reaction) [41].

On the basis of clinical manifestations and laboratory data, the affected dogs were classified as SIRS positive. The SIRS criteria used in the study were established on the basis of the criteria proposed by Purvis and Kirby (1994) [42] and Welzl [43]. The animal was classified as SIRS positive if two or more of the following 4 criteria were fulfilled: body temperature higher than 39.5 or lower than 38°C, heart rate higher than 160 beats/min, respiration rate higher than 20 breaths/min and WBC count lower than 6 x 10^9^/L or more than 12 x 10^9^/L or more than 10 percent band neutrophils. None of the dogs selected for this study presented organ dysfunction at the time of blood extraction.

Group 2 (healthy controls) consisted of 10 dogs of different breeds and sexes, aged from 1 to 10 years. These dogs were considered healthy based on physical examination as well as hematological and biochemical data, and they attended the hospital to receive a prophylactic dose of imidocarb dipropionate (6 mg/kg) as a preventive measure against babesiosis upon their owners’ request. The protocol was approved by the Ethics Committee for Animal Experimentation, Faculty of Veterinary Medicine, University of Zagreb, Croatia (Permit No: 251–61–01/139–12–2). All dogs were admitted to the Clinic for Internal Diseases, Faculty of Veterinary Medicine, University of Zagreb, Croatia and written owner consent was obtained for all dogs included in this study as part of a routine clinical protocol. Owners were informed about the experimental use of the specimens.

All serum samples from dogs were screened for simultaneous qualitative detection of circulating *D*. *immitis* antigen and antibodies, both immunoglobulin G and M, to *E*. *canis*, *B*. *burgdorferi sensu lato* and *A*. *phagocytophilum* with the SNAP 4Dx test (IDEXX Laboratories, Hoofddorp, The Netherlands). The same samples were further qualitatively tested for antibodies to *L*. *infantum* with SNAP *Leishmania* (IDEXX Laboratories). Both tests were performed according to the manufacturer’s instructions. All samples were negative for the aforementioned vector-borne pathogens.

### Blood sample analysis

Blood samples for analysis from groups 1 and 2 were collected from the cephalic vein on the day of admission, and before the administration of imidocarb dipropionate (Imizol). The samples were placed in tubes with ethylenediaminetetraacetic acid (EDTA) for haematological analysis and for establishing the diagnosis. Blood smears and PCR were performed from samples taken at admission. The samples for biochemical analysis were collected in tubes with no anticoagulant and were centrifuged at 1200 × g. A portion of the obtained serum was used for establishing biochemical profiles while the remainder was stored at –80°C until used for proteomic analysis. Complete blood count was analyzed using an automatic haematology analyzer, Horiba ABX (Diagnostics, Montpellier, France). Biochemical profiles were generated according to standard methods using an automated biochemistry analyzer (Olympus AU 600, Olympus Diagnostica GMBH).

### Protein incubation and digestion

We performed filter aided sample preparation (FASP) using 10kDa cut-off filters. 10 control serum samples and 10 serum samples from dogs with babesiosis (7 μl per sample) were incubated for 30 min in 20% acetonitrile / 80% 50mM ammonium bicarbonate (18μl) at room temperature to allow the release of peptides bound to albumin in the presence of phosphatase inhibitor cocktail set V 50x (Merck Millipore, MA USA) diluted 50-fold. 5μl of 200mM DTT was added to each sample and reduction was allowed to proceed for 30 min at room temperature. After reduction, 20μl of 200mM IAA was added and samples were incubated for 30 min at room temperature.After alkylation, 20μl of 200 mM DTT was added to reduce any excess IAA and samples were incubated for 30 min at RT. Digestion was performed by adding 1μg of Trypsin Gold to each sample (trypsin-to-protein ratio 1:100, w/w). Digestion was allowed to proceed overnight at 37°C and peptides were dissolved in 80% acetonitrile and 2% formic acid. Peptides were kept at -20°C prior to phosphopeptide analysis.

### Peptide mixture desalting

For identification, prior to LC-MS/MS analysis, enriched tryptic peptides were desalted using ZipTip C18 (Merck Millipore, Darmstadt, Germany) according to the manufacturer’s procedure and vacuum-dried.

### TMT labeling of pooled serum samples

Sera from 10 healthy dogs were pooled. Separately, sera from 10 *Babesia*-infected dogs were pooled and both pools were mixed with protease inhibitors. The protein concentration of healthy and *Babesia*-infected pools was determined by BCA assay. Peptides were labelled using two different tags from a TMTsixplex isobaric label reagent set (*m/*z 128 and *m/*z 129 isobaric tags) according to the manufacturer’s procedure (Thermo Scientific, Waltham. MA, USA) with some modifications. In short, 35 μg of total protein was diluted to a final volume of 50 μl using 0.1 M triethyl ammonium bicarbonate (TEAB, pH 8.5). A volume of 2.5μl of 200 mM DTT was added to each sample followed by incubation for 1 h at 55°C. Alkylation was performed by adding 2.5 μl of 375 mM IAA and incubating for 30 min at room temperature in the dark. Proteins were acetone-precipitated (overnight at -20°C) and centrifuged at 8000 x*g* for 10 min at 4°C. Protein pellets were reconstituted in 50 μl of 0.1 M TEAB and trypsin digested (trypsin-to-protein ratio 1:30, w/w) at 37°C overnight. TMT reagents were dissolved according to manufacturer recommendations. For the labelling reaction, 18 μl of freshly prepared TMT reagent was added to each sample and incubated for 1h at room temperature. The reaction was quenched by adding 8 μl of 5% (w/v) hydroxylamine HCl solution (incubation for 15 min). Finally, equal amounts of differentially TMT-labelled peptides were combined into the final sample. 10 μg of the final sample was vacuum-dried and stored at -80°C for LC-MS/MS analysis. The remaining sample was used for phosphopeptide enrichment.

### Phosphopeptide enrichment

Pierce^TM^ phosphopeptide enrichment containing TiO_2_ magnetic microbeads (Thermo Fischer Scientific, Waltham, MA, USA) was used to enrich phosphopeptides. Briefly, 30 μl of bead mixture for every sample was equilibrated with binding buffer by washing the microbeads three times on a magnetic support. Serum peptides obtained after digestion were added to the microbeads and resuspended by repeated pipetting. After a short incubation (2 min) at room temperature, non-bound peptides were washed off from the microbeads by adding binding buffer and washing on a magnetic support (3 times). After binding, washing buffer was added quickly and then removed completely. Elution buffer (30 μl) was added, the sample resuspended repeatedly and allowed to stand for 10 min at room temperature after which eluted phosphopeptides were collected, quantified using nanodrop and kept at -20°C for further LC-MS/MS analysis.

### LC-MS/MS analysis

Peptides were reconstituted in 0.1% (v/v) FA and 1μg was separated on an Ultimate 3500 RSLS nanoflow system (Dionex) before online ESI-MS/MS analysis with a Q Exactive Plus mass spectrometer (Thermo Scientific). For phosphoprotein ID, direct injection onto a PepMap RSLC C18 analytical column (15 cm x 75 μm) with a linear gradient 5–35% mobile phase B (0.1% FA in 80% ACN) over 180 min at a flow rate of 300 nL /min was used to separate peptides. For TMT-based relative quantification, peptides were loaded onto a cartridge trapping column (C18 PepMap100, 300 um x 5 mm) using a 15 μl/min flow rate and desalted for 12 minutes by mobile phase C (1% ACN in 0.1% FA) before separation on a PepMap RSLC C18 (50 cm x 75 μm) analytical column.

The MS instrument was operated using a Nanospray Flex ion source with a SilicaTipemitter (New Objective). The ionisation voltage was set to 1.9 kV and the ion transfer capillary temperature to 275°C. MS was operated in positive ion mode using FT HCD MS2. Full scan FTMS spectra were acquired in the range from *m/z* 350 to *m/z* 1900 with a resolution of 70000. The maximum injection time for the FTMS full scan was set as 110 ms reaching an AGC target value of 3x10^6^. The eight most intense precursor ions were chosen for HCD fragmentation with a resolution of 17500 using an AGC target of 2x10^5^. The step collision energy was set to 28% and 35% NCE, respectively. For relative quantification, as a third step 40% NCE was used. A ± 1.6 Da isolation window was applied to isolate precursor ions with a dynamic exclusion of 30 s. Minimal PSM number to confirm the presence of a phosphopeptide was set at 2. The mass spectrometry proteomics data have been deposited to the ProteomeXchange Consortium via the PRIDE partner repository with the dataset identifier PXD010894.

### Data analysis and phosphorylation/kinase database search

Proteome Discoverer (version 2.0.0.802) software performing SEQUEST search of *Canis lupus familiaris* fasta files (downloaded from NCBInr database 2016/10/13) was employed for data analysis. Static peptide modification included carbamidomethylation (C), dynamic oxidation (M) and phosphorylation (S, T and Y). One trypsin missed cleavage was allowed. Precursor tolerance and ion fragment tolerance were set at 10 ppm and 0.05 Da, respectively. Confidence levels were set to 1% FDR (high confidence) and 5% FDR (middle confidence). On average 90% of the identified phosphopeptides showed high confidence (FDR 1% or lower).

Proteome Discoverer node ptmRS was used for analysis and mapping of peptide/protein phosphorylation sites. ptmRS Best Site Probabilities displays the most likely positions of the modifications and their site score for each PSM. For each modification site, this value is an estimate of the probability (0–100%) that the site is truly modified. Any ptm*RS* site probabilities above 75% indicate that a site is truly modified. In multiply phosphorylated peptides ptmRS site probabilities often drop below 50% although the same phosphorylation site was observed in the same monophosphorylated peptide at higher ptmRS probabilities. In the present study only phosphorylation sites reaching 95% or higher ptmRS probabilities were considered.

For peptide groups, this column shows the best site probabilities of the first PSM. ptmRS *Modification* Site Probabilities: Displays the modification site probabilities for all possible modification positions for a single modification. In the column title, *Modification* is replaced by the name of the actual modification, for example, Phosphorylation Site Probabilities [44]. Phosphorylation sites detected were matched to phosphorylation sites found in PhosphoSitePlus database (https://www.phosphosite.org/homeAction) [45]. Assignment of putative kinases and corresponding score calculation for the experimentally observed phosphorylation sites was performed using NetPhos 3.1 server (http://www.cbs.dtu.dk/services/NetPhos/)[46]. Weblogo sequence alignment analysis was performed using protein sequences from 10 mammalian species by means of web server https://weblogo.berkeley.edu/logo.cgi [47].

## Results

**Fig 1:**
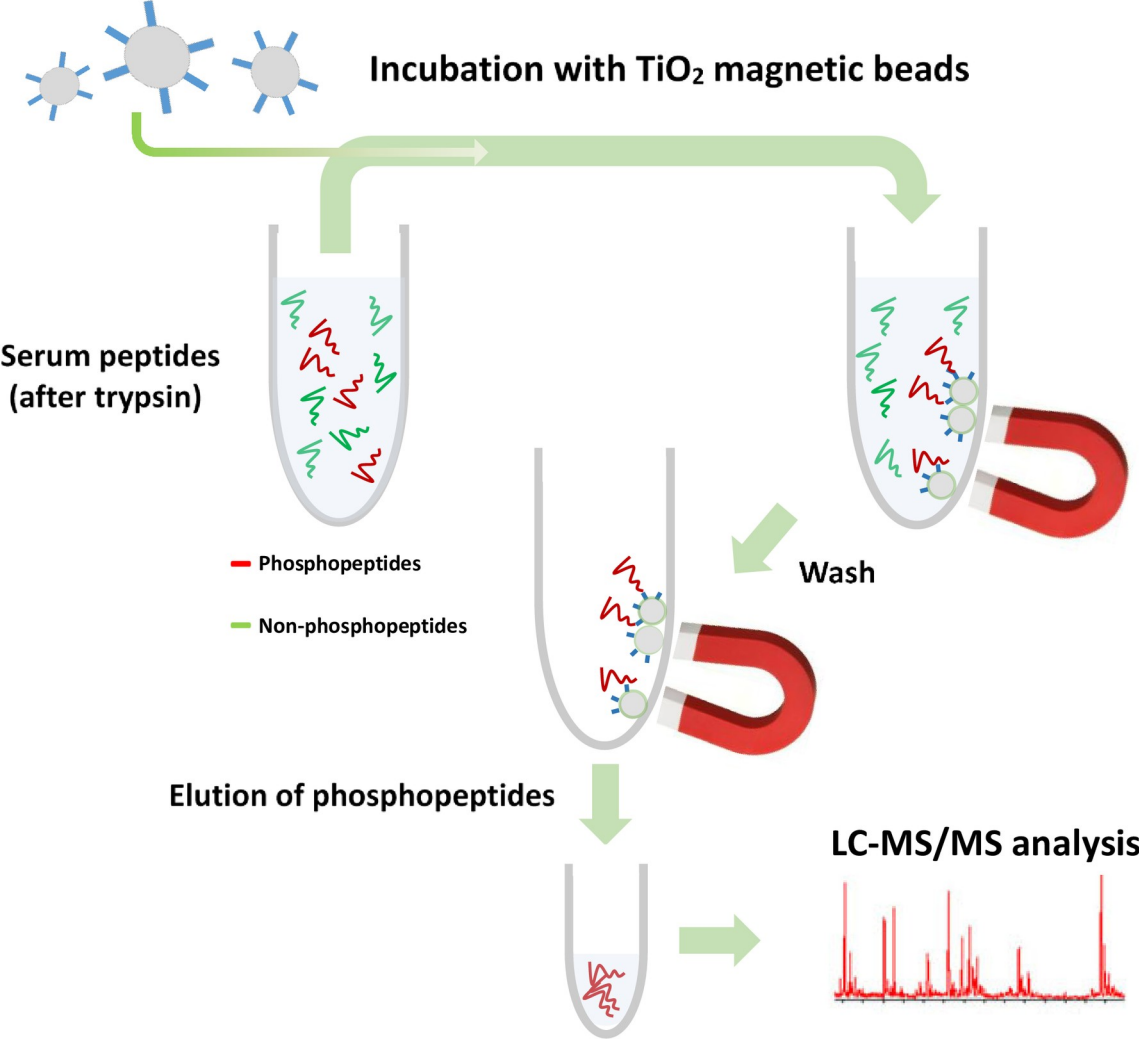
Phosphopeptide enrichment scheme. Magnetic TiO_2_ beads were used after in-solution filter aided sample preparation *(*FASP*)* and (phospho) peptide extraction. Samples containing (phospho) peptides in 80% acetonitrile and 2% formic acid were mixed with the beads and several washes were applied using magnets to retain beads at the bottom of each tube. In a final step, enriched phosphopeptides were eluted from beads by the addition of high pH acetonitrile solution.

Canine serum phosphoproteins were analyzed qualitatively and quantitatively after phosphopeptide enrichment by means of metal oxide affinity using TiO_2_ magnetic microbeads. Fig 1 depicts the enrichment process employed in this study.

Phosphopeptides were enriched according to their affinity for TiO_2_ after several binding steps followed by extensive washing and a final elution step. After optimizing sample preparation and LC-MS/MS protocols as well as data processing, we observed qualitative changes in phosphoprotein content of individual serum samples. Additionally, we performed a preliminary quantitative analysis using TMT- labeling, along with LC-MS/MS, of a serum pool composed of 10 control samples and a babesiosis serum pool composed of 10 serum samples from *Babesia*-infected dogs.

**Fig 2:**
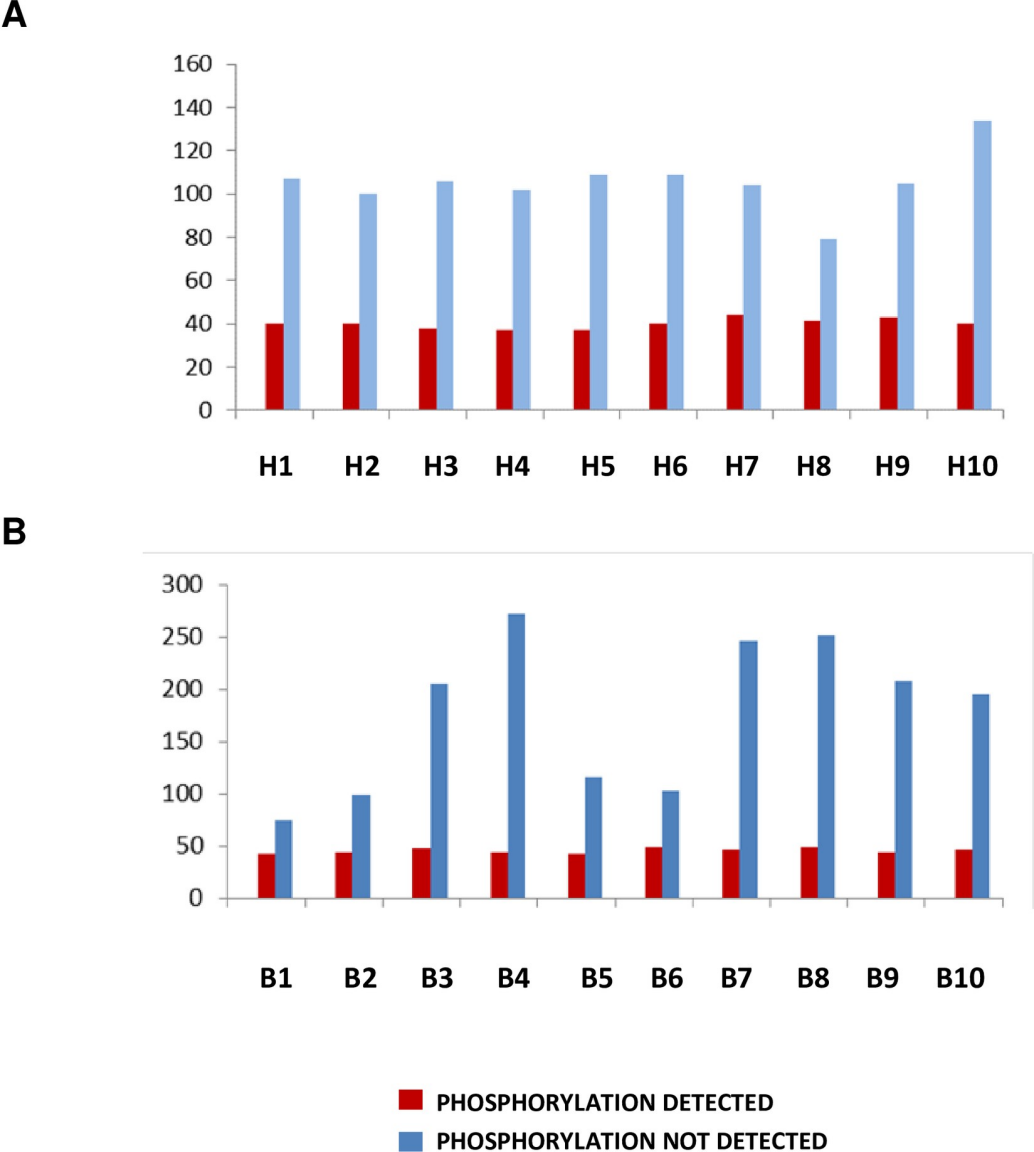
**Composition of enriched peptides** expressed as the number of proteins which corresponding peptides were phosphorylated (red) and the number of peptides from proteins with no phosphorylation detected (blue) for each sample analyzed. A, healthy dogs. B, dogs suffering from babesiosis.

After LC-MS/MS analysis of the enriched phosphopeptides, we found approximately 50 proteins with detected phosphorylation sites and a variable number of non-phosphorylated proteins (Fig 2). The number of non-phosphorylated peptides corresponded to around 100 proteins in control sera whereas it reached 250 in babesiotic sera. A moderate positive correlation was found in sera from babesiosis cases between the number of bound non-phosphorylated peptides and alkaline phosphatase concentrations (not shown).

Qualitative results showed approximately 89 phosphorylation sites in about 50 proteins: 64 phosphoserines (71.9%), 15 phosphothreonines (16.8%) and 10 phosphotyrosines (11.2%) (Table 1). The presence or absence of the detected phosphoproteins (with phosphorylation site detected) was tested for a series of 10 control and 10 babesia sera samples.

**Table 1:** Qualitative analysis of phosphoproteins with phosphorylation site detected. Phosphorylation sites within peptide sequences and number of samples in which corresponding phosphoprotein was detected after analysis of the fraction bound to TiO_2_ in healthy (H) and babesiosis-affected dogs (B). #Unique and #PSM correspond to number of unique peptides and spectral counts for a comparison of 10 healthy samples vs. 10 babesiosis samples respectively. NetPhos 3.1 scores and corresponding predicted kinases are shown for each phosphorylation site. Kinases: CaMKII: Ca^2+^/calmodulin-dependent protein kinase II; CKI: Protein kinase CK1; CKII, Protein kinase CKII; DNAPK: DNA-dependent protein kinase; EGFR: Epidermal growth factor receptor; GSK3: Glycogen synthase kinase 3; INSR: Insulin receptor; p38MAPK: p38 mitogen-activated protein kinase, PKA: Protein kinase A; PKC: Protein kinase C; PKG: Protein kinase G; RSK, ribosomal s6 kinase. P, detected only in pools.

Protein	Phosphosite	Peptide	H	B	#Unique peptides	#PSM H-B	Accession		
α-2- antiplasmin **	S450	kEQQDsPDDRDYFQNr / 0.508 CKII	5	10	2–3	41–41	73967363		
	S480	lAPPsEEDYPQLHSPk / 0.498 CKI	5	5	2–3	41–41			
	Y484	lAPPSEEDyPQLHSPk0/ 0.504 INSR	5	5	2–3	41–41			
α-2-HS-Glycoprotein**	S138	cDSSPDsAEDVRk / 0.526 CKI	10	10	11–14	2664–1570	545553759		
	S323	hAFMGVAsVESASGEAFHVGk / 0.458 CaMK-II	10	10	11–14	2664–1570			
	S326	hAFMGVASVEsASGEAFHVGk/ 0.484 CaM-II, 0.440 GSK3	10	5	11–14	2664–1570			
	S328	hAFMGVASVESAsGEAFHVGk/ 0.428 GSK3 ,	10	0	11–0	2664–0			
Chromogranin A*	S190	hPDsQAEEDSEGLSQGLVDTEk/ 0.602 DNAPK, 0.595 CKII	4	1	1–1	4–1	244539517		
Apolipoprotein A1**	S211	eGGGAsLAEYHAr / 0.645 PKA, 0.482 CKII	10	10	31–39	459–1005	928133662		
	S220	eGGGASLAEYHARAsEQLSALGEk / 0.619 PKA	5	0	31–0	459–0			
Coagulation factor V**	S931	wHLVsEKGSYEIVPDAEDMAVDk / 0.513 PKC	P	P	1	3	545504920		
	S935	wHLVSEKGsYEIVPDAEDMAVDk / 0.472 GSK3 ,	P	P	1	3			
	Y936	wHLVSEKGSyEIVPDAEDMAVDk / 0.508 EGFR k	P	P	1	3			
Complement factor H**	S883	sSIFsEEIEETSKPk / 0.629 CKII, 0.571 CKI	10	10	3–14	37–48	74005944		
DNA directed RNA* polymerase subunit RPA43	S300	kKHQEVQDQDPVFQGSDSsGYQSDHk / 0.857 PKC, 0.524 CKII	0	2	0–1	0–6	Q3B726		
Fibrinopeptide A/Fibrinogen α **	T20	tNSKEGEFIAEGGGVr / 0.491 CKII 0.481 cdc2	9	3	1–1	46–11	73978329		
	S22	tNsKEGEFIAEGGGVr / 0.563 CKI 0.458 CaMKII	9	3	2–1	46–11			
Fibrinopeptide B/Fibrinogen β **	Y31	hyYDDTDEEEr / 0.377 INSR	4	5	1–1	30–53	545524893		
	Y32	hYyDDTDEEEr / 0.380 INSR ,	0	2	1–1	30–53			
	T35	hYYDDtDEEEr / 0.734 CKII	4	5	1–1	30–53			
Fibrinogen γ **	S128	yEALVGsHESNIr / 0.477 cdc2	P	P	2	4	73977992		
Fibronectin**	S2384	rTNTNVNCPIECFMPLDVQADREDsRe 0.559 RSK	10	10	8–5	58–42	P02751		
Immunoglobulin heavy chain variable region **	T47	rLSCVASGFtLSDYGLSWVr/ 0.476 CaM-II	P	P	1	3	208342066		
Insulin-like growth factor binding protein 3 *	S201	vDYESQSTDTQNFsSEYk/ 0.521 CKII, 0.519 CKI	3	0	1–0	6–0	359321488		
Inter-alpha-trypsin inhibitor heavy chain H2 (ITIH2)**	S60	sVFGEsGEVMEEADQVTLYSYk / 0.589 CKII	P	P	11	17	73949158		
Histidine-rich glycoprotein**	S464	qGQGPPPQHsEEr / 0.448 GSK3	5	5	3–3	70–42	545553762		
Kanadaptin*	T625	lQQEtELEEAVQDTRPPTDLMCSKETk / 0.714 CKII	2	0	1–0	4–0	545527407		
	T634	lQQETELEEAVQDtRPPTDLMCSKETk / 0.495 cdc2	2	0	1–0	4–0			
Kininogen 1 X1**	T326	etMCSKESNEELAESCQINk / 0.600 PKC	10	10	7–12	48–38	345796419	
	S332	eTMCSKEsNEELAESCQINk / 0.660 CKII ,	10	0	9–0	230–0	
X2**	T326	etMCSKESNEELAESCQINk / 0.600 PKC	10	10	3–10	35–147	57109938		
	S332	esNEELAESCQINk / 0.590 SRC	10	0	6–0	164–0			
Kinesin-like KIF26A*	S1726	lGRKPsLPGQWVDLPPPLAGSLk / 0.823 PKA, 0.542 PKG ,	0	2	1–1	1–2	Q9ULI4		
Serum albumin**	T267	vHtECCHGDLLECADDRADLAk / 0.451 CKII	10	10	3–4	50–73	P02768-1		
Serum albumin X1**	S494	mSCADDFLSVVLNRLCVLHEKTPVsEr/0.743 PKC	P	P	2	3	545520919		
Serum amyloid A**	S101	fGDSGHGAEDsKADQAANEWGr / 0.461 cdc2 EWGRSGKDPN HFRPAGLPDK Y	0	10	0–8	0–382	545536980		
Sulfhydryl oxidase**	S398	hNLDHsQETAEAQEVLQAIr / 0.649 DNAPK	6	9	1–1	9–24	928139154		
	T401	hNLDHSQEtAEAQEVLQAIr / 0.649 DNAPK	6	9	1–1	9–24			
Golgi membrane protein 1**	S276	gETNEIQVTsEEEPQr / 0.658 CKII ,	P	P	1	2	928125053		
Vitronectin**	Y75	rGDVFTMPEDEyTVYDDGEEk / 0.634 SRC	P	P	6	9	73966959		

*, False Discovery Rate 5%

**, False Discovery Rate 1%

There was an important difference in the presence of serum amyloid A (SAA), an acute phase protein, in samples from babesiotic dogs in comparison to controls. A considerable number of phosphosites was detected for SAA in diseased samples with serine (S) 101 phosphorylated in all controls but in none of the sera from babesiotic dogs. Fibrinogen β was found to be phosphorylated at tyrosine (Y) 32 only in 2 samples from infected animals and in none of the controls. Kininogen-1, an important protein in coagulation, was phosphorylated at threonine (T) 326 and serine (S) 332 in all samples. Another protein with a relatively high number of phosphorylation sites was α-2-antiplasmin, which was phosphorylated at S514 in 10 samples from diseased dogs vs. 5 control samples.

On the other hand, a higher number of phosphorylated proteins was found in control samples in case of ApoA1 at S220 (5 controls vs. none in babesiosis samples), Chromogranin A at S190 (4 controls vs. 1 babesiosis) and Fibrinogen α at S22 (9 controls vs. 6 babesiosis). Finally, multiple phosphofibronectin isoforms were detected in this analysis in all of the samples (Table 1).

**Fig 3:**
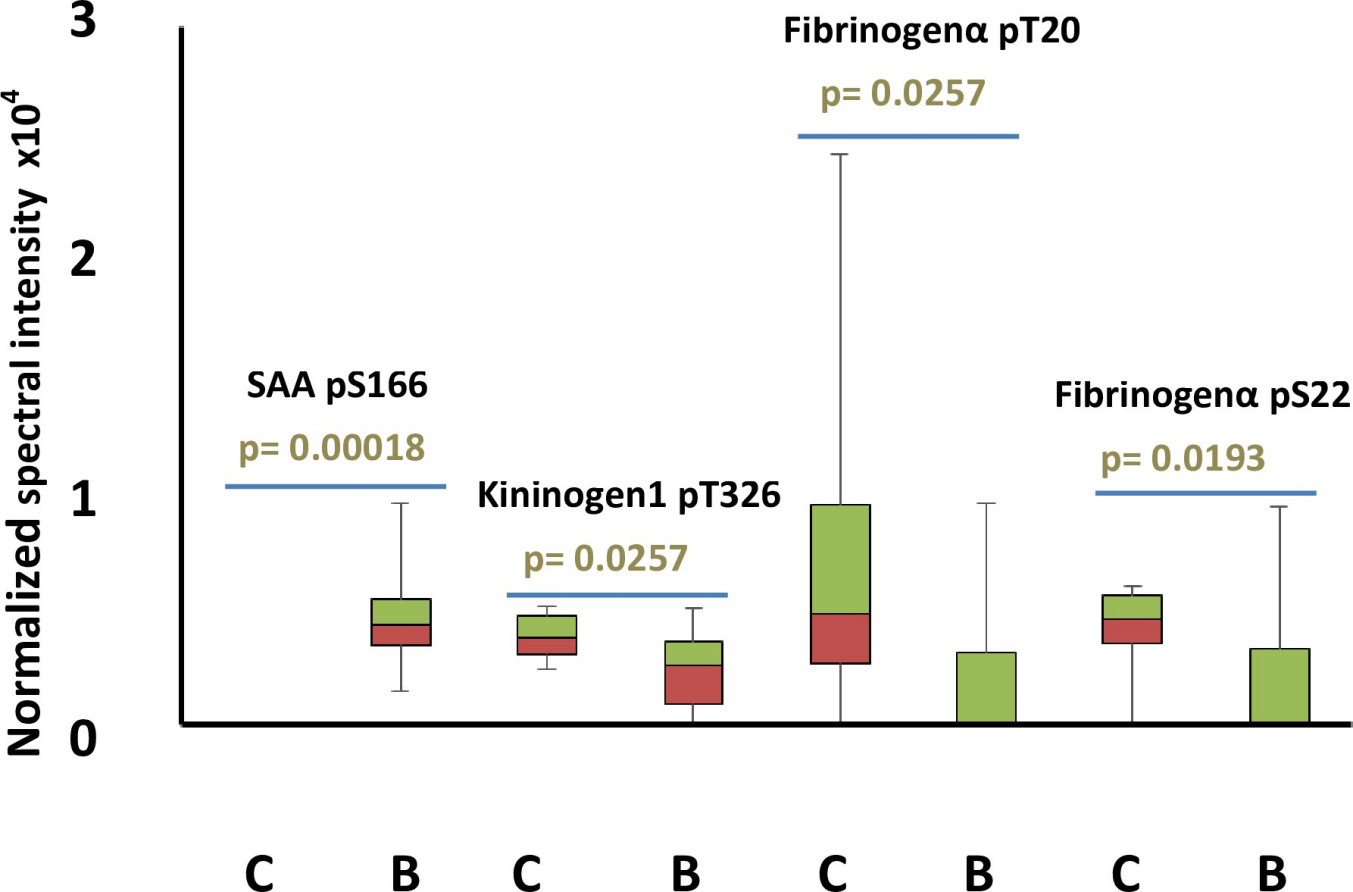
Box plot of normalized spectral intensities of significantly varying phosphopeptides found in controls (n = 10) vs. babesiosis (n = 10). Serum amyloid A (SAA) phosphorylated at serine 166 (pS166), kininogen 1 phosphorylated at threonine 326 (pT326), Fibrinogen α phosphorylated at threonine 20 (pT20) and at serine 22 (pS22) are shown for samples from controls (C) and samples from dogs suffering from babesiosis (B). p-value for the comparison C vs. B was calculated using Mann-Whitney test and it is shown for each phosphopeptide. Sequences corresponding to each peptide can be found in Table 1. Normalized phosphopeptide spectral intensity = Spectral intensity of phosphorylation site/Total spectral intensity of the sample.

Out of all phosphopeptides detected in individual samples, 3 showed significantly different spectral intensities in babesiosis-affected dogs compared to controls. Serum amyloid A phosphorylated at S101 was the most significant increase (p = 0.00018) in phosphopeptide levels according to Mann-Whitney test (Fig 3), since it was not detected in any of the control samples but appeared in all babesiosis samples.

Kininogen1 phosphorylated at T92 (p = 0.0257) and fibrinogen α phosphorylated at T20 and S22 (p = 0.0257 and p = 0.0193, respectively) showed significant decreases in babesiosis samples. S1 Table shows clinical values of dogs engaged in this study as well as normalized intensities of phosphopeptides with significantly (Mann-Whitney test p-value<0.05) different intensities in controls and babesiosis samples.

**Fig 4:**
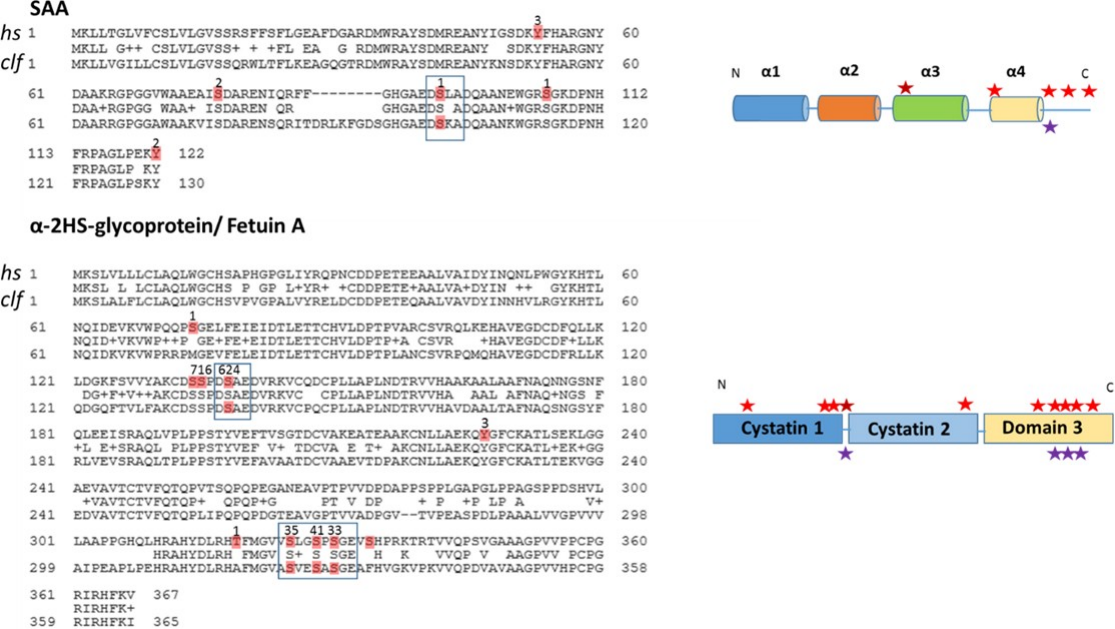
Comparison of phosphorylation sites in human and dog for representative proteins. **Top and bottom left,** serum amyloid A and α-2HS-glycoprotein / fetuin A sequence alignments for *homo sapiens* (*hs*) and *canis lupus familiaris* (*clf*) highlighting the phosphorylation sites found in databases and experimentally detected phosphorylation sites found in canine samples (both in red). Number of references supporting each phosphorylation site is included on each site. Sites common for human and dog are shown in boxes. **Top and bottom right,** schematic drawing of respectively SAA and α-2HS-glycoprotein / fetuin A domain structure and location of phosphorylation sites for human (red and dark red for the phosphorylation site with the highest number of references) and dog (violet) orthologs.

Sequence alignment of canine SAA and α2-HS-glycoprotein with human orthologs revealed some conserved phosphorylation sites according to PhosphoSitePlus database search (Fig 4).

Phosphorylation sites detected in the present analysis were compared with the phosphosite plus database data for human, mouse, rat and cow and conserved sites are listed in Table 2.

**Table 2:** Phosphorylation sites in PhosphoSitePlus database vs. detected phosphorylation sites in canine serum protein orthologs.

	Human	Mouse	Rat	Cow	Dog	Novel
**A-2HS-glycoprotein / Fetuin A**						
S74	+					
S134	+					
S138	+	+	+		+	
S325	+	+	+	+	+	
S328	+	+	+	+	+	
S330	+	+	+	+	+	
**Apolipoprotein A1**						
S166		+	+				
S191			+			
S211					+	*
Y216		+				
T221		+			+	
S225		+				
T226		+				
**Albumin**							
T263	+	+	+	+		
T267					+	*
**Arrestin beta 1**						
T410	+	+	+			
S412	+	+	+		+	
**Complement factor H**						
S883		+			+	
**Fibrinogen α**						
T20					+	*
S22	+				+	
**Fibriniogen β**						
Y31					+	*
Y32					+	*
T35					+	*
S58	+					
Y71	+					
**Fibrinogen γ**						
Y94	+					
S128					+	*
Y140	+					
**Histidine-rich glycoprotein**						
S438		+	+		+	
**ITIH2**						
S60	+	+			+	
**Kininogen 1**						
T326	+				+	
T327	+				+	
S329	+					
S332	+					
T337	+					
T342	+					
**SAA**						
Y53	+	+				
S77	+					
S94	+				+	
S106	+					
Y112		+				
Y122	+					

**Fig 5:**
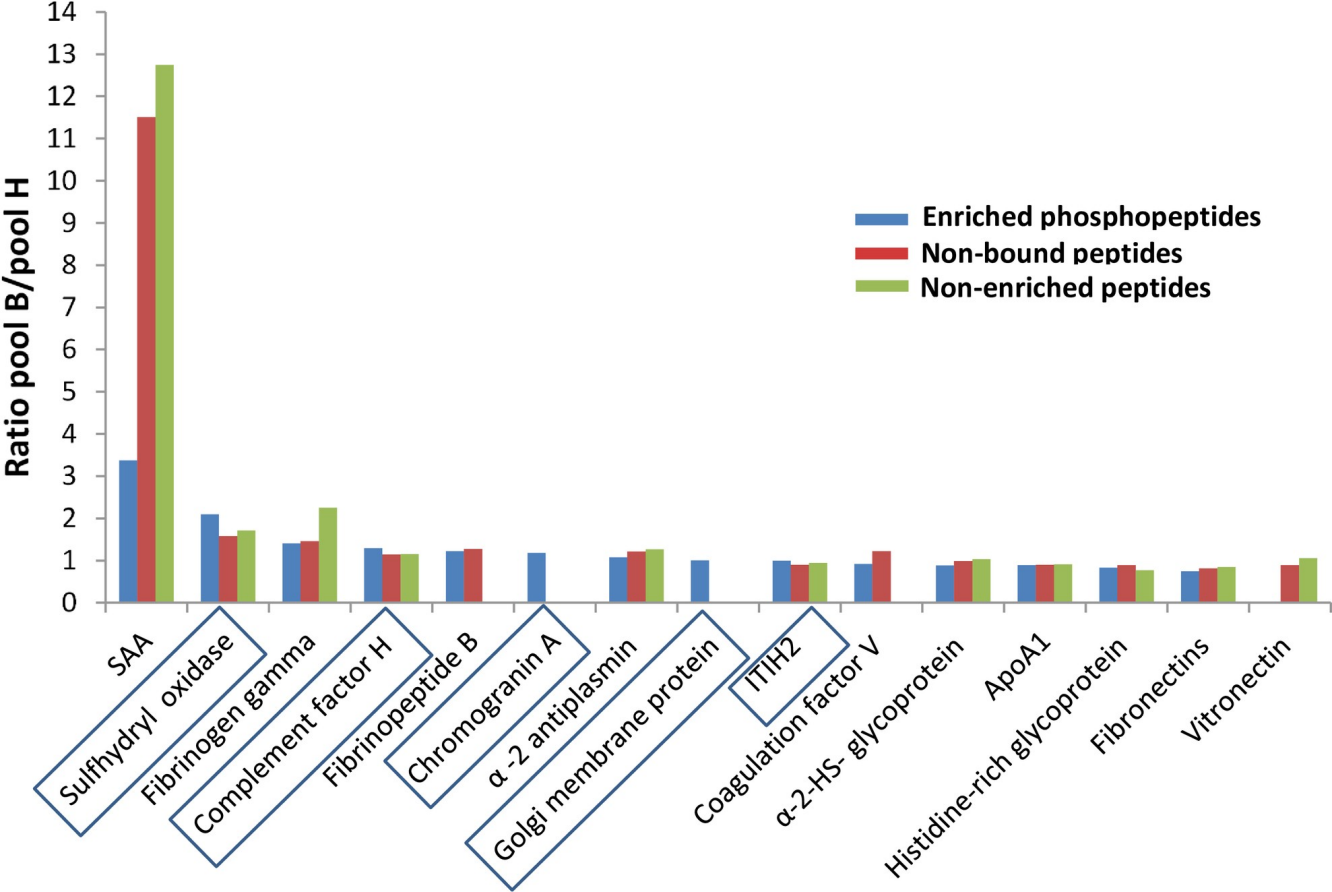
Intensity ratios pool B/pool H for enriched phosphopeptides compared to pool B/ pool H ratios of total peptide intensities in non-bound and in non-enriched pools. B/H ratios of phosphopeptide intensity in enriched pools for the corresponding protein is indicated in blue. B/H ratios for intensities of peptides not bound to TiO_2_ are shown in red and ratios for peptides observed in pools with no enrichment are shown in green. For some proteins not a single peptide was detected in non-bound or non-enriched (direct) pools. Proteins with higher ratios of enriched phosphopeptides than non-bound and non-enriched peptides are highlighted.

TMT-labeling-based quantitative analysis of the control and babesiosis pools revealed often, most notably in the case of SAA, that the global level of a protein (phosphorylated and non-phosphorylated) was several fold higher in babesiosis pool (pool B) (Fig 5). Sulfhydryl oxidase, Complement factor H and inter-alpha-trypsin inhibitor heavy chain 2 (ITIH2) were the only proteins with a higher poolB/poolH ratio for enriched phosphopeptides than for non-bound and non-enriched peptides. Serum amyloid A (SAA) showed the highest pool B/ pool H ratios of all proteins (Fig 5).

**Fig 6:**
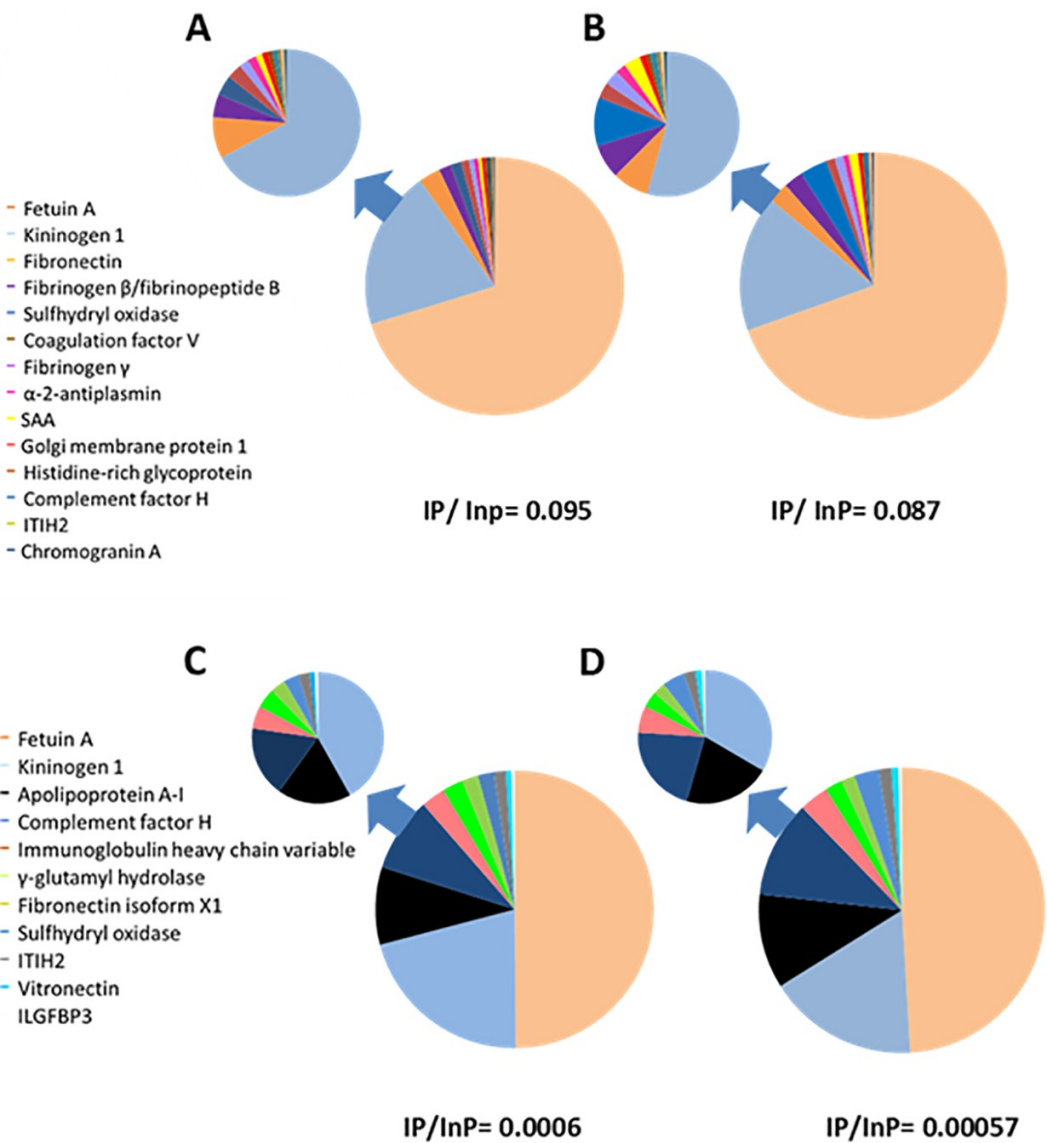
Relative intensities of TMT-labeled phosphopeptides with respect to total phosphopeptide intensity found in enriched and non-enriched fractions of dog serum pools. A, Pool composed of sera from 10 healthy dogs, B, Pool composed of 10 sera from babesiotic dogs. Intensities of TMT-labeled phosphopeptides are shown in C, healthy pool and D, babesiosis pool. In all cases, all phosphoproteins except alpha-2-HS-glycoprotein/fetuin-A are shown separately in the inset. IP = Intensity of phosphopeptide, InP = Intensity of non-phosphopeptide.

After summarizing quantitative data for the top TMT-labelled phosphoproteins detected, our data show that alpha-2-HS-glycoprotein / fetuin-A is the most abundant phosphoprotein present in dog serum both in enriched and non-enriched pools (Fig 6) with around 50–70% of the total intensity corresponding to enriched phosphopeptides and no variation when comparing control and babesiosis pools.

Second in abundance was kininogen-1 with 19.9% of total intensity of TMT-labelled phosphopeptides in the control pool vs. 16.5% in the babesiosis pool. Other remarkable changes observed were serum amyloid A (0.4% in the control pool vs. 1.1% in the babesiosis pool), sulfhydryl oxidase (1.3% to 3.3% increase), fibrinopeptide B (1.5% to 2.3%), fibrinogen gamma (0.6% to 1.02%) and coagulation factor V (0.99% to 1.06%), while most other proteins remained at similar levels (Fig 6). Data before and after enrichment allowed us to evaluate the enrichment yield. Phosphopeptide intensity/total peptide intensity ratio for pool H was 0.0006 before enrichment and 0.095 after enrichment, whereas for pool B it was 0.00057 before and 0.087 after enrichment (Fig 6), indicating a 150-fold phosphopeptide enrichment.

**Fig 7:**
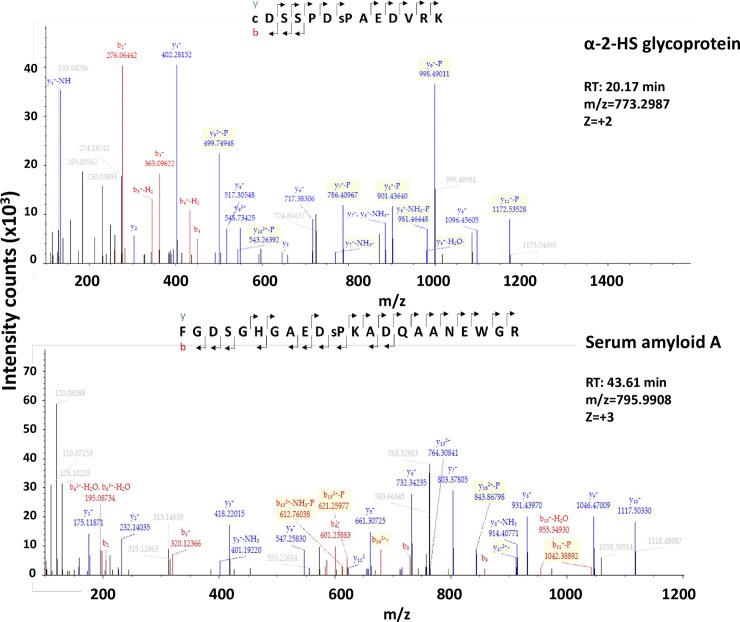
Spectra of representative phosphopeptide. A, Alpha-2HS-glycoprotein /Fetuin A. **B,** Serum amyloid A.

Fig 7 shows spectra corresponding to two of the most representative phosphopeptides found in this analysis, serum amyloid A and α-2HS-glycoprotein/fetuin A.

**Fig 8:**
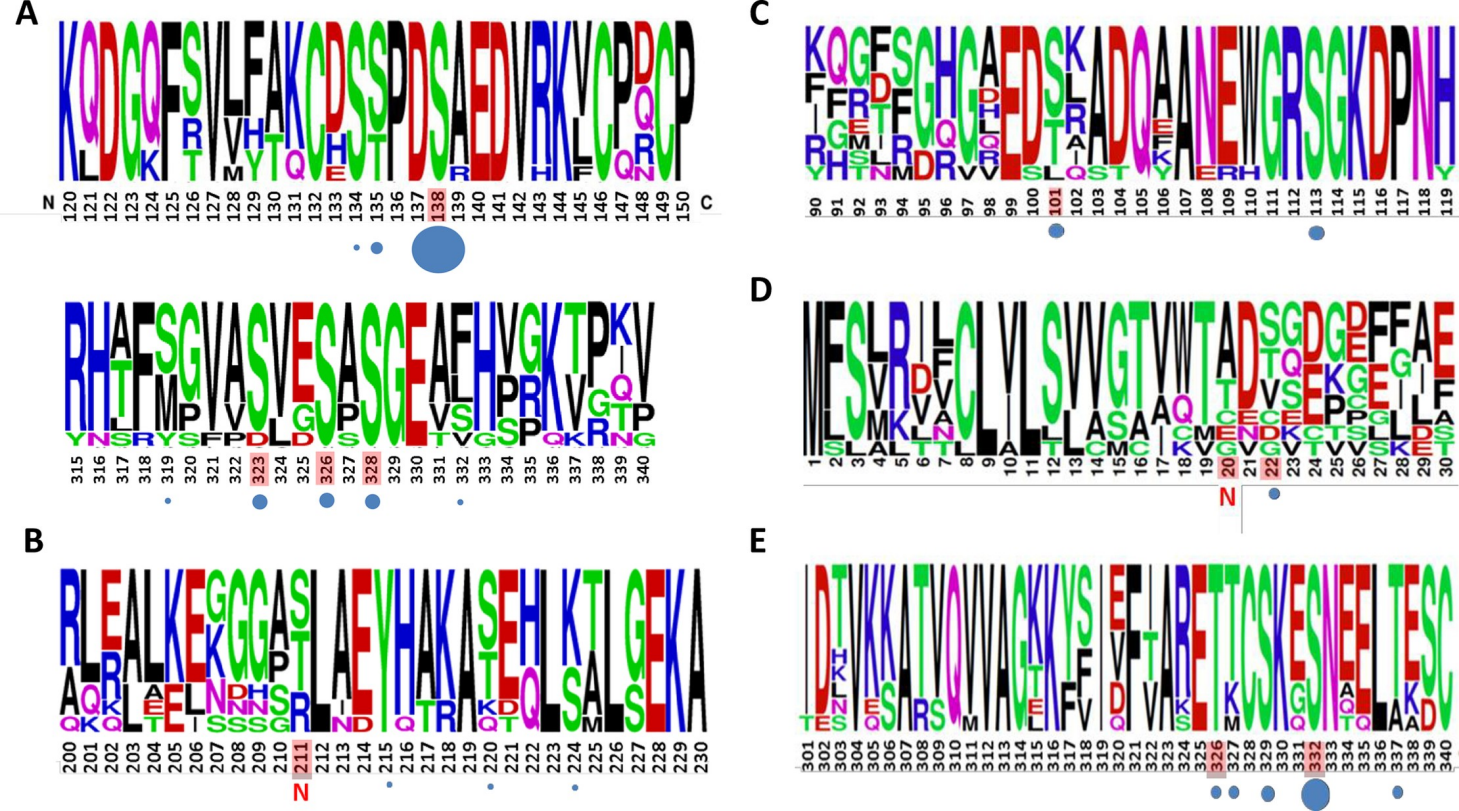
WebLogo analysis of of phosphorylation sites Sequence conservation among 10 mammalian species (Human, Mouse, Rat, Cow, Cat, Chimpanzee, Dog, Guinea pig, Pig, Sheep) and phosphorylation conservation. Frequency distribution of aminoacid residues of surrounding phosphorylation sites are shown for: A, α-2-HS-Glycoprotein; B, Serum amyloid A (SAA); C, Apolipoprotein A1; D, Fibrinogen α and E, Kininogen 1. Circle size under sequences is proportional to the number of studies in which the corresponding phosphorylation site was observed (in PhosphoSitePlus databases). In red, phosphorylation sites detected in dog serum. N highlights novel sites.

Amino acid sequences of selected phosphopeptides observed in the present study were submitted to weblogo analysis in order to determine sequence conservation and to allow comparison with previously reported phosphorylation sites (Fig 8).

S2 Table shows MS parameters of a representative selection of phosphopeptides detected in individual serum samples and in TMT-labeled serum pools. S1 Fig shows MS spectra corresponding to representative phosphopeptides detected in the present analysis.

## Discussion

We have focused our study on the relatively new field of serum phosphoproteomics combining individual sample qualitative/quantitative label-free and TMT label-based quantitative pool proteomic analysis. The present analysis is to our knowledge the first to accomplish a preliminary characterization of phosphorylation sites of major serum proteins in dogs to yield an initial characterization of the canine serum phosphoproteome. Additionally, our quantitative study aimed to perform an initial assessment of the detectable modifications of serum phosphoprotein abundancies induced by babesiosis.

Our data suggest that the increase in concentration of acute phase proteins, proteins released from lysed erythrocytes due to hemolysis, and apolipoproteins could lead to an increase of unspecific binding of these proteins to TiO_2_ magnetic beads due to their apolarity as preciously observed [48, 49]. Despite phosphopeptide enrichment yielded a 150-fold enrichment, the number of identified phosphoproteins is far from that obtained in cell lysates [50]

Our analysis found several phosphorylation sites not included in current protein phosphorylation databases such as PhosphoSitePlus, PhosphoElm 9.0, PHOSIDA, dbPAF, dbPTM 3.0 Uniprot and others. These databases contain a maximum of a half million phosphorylation sites found in around 50,000 proteins from different species, including *Homo sapiens*, *Mus musculus*, *Rattus norvegicus*, *Drosophila melanogaster*, *Caenorhabditis elegans*, *Sacchamomyces cerevisiae and Schizosaccharomyces pombe* [12], but very scarce information for *Canis lupus familiaris*. Computational analysis of phosphorylation sites in protein databases has shown that kinases hold the highest degree of conservation among the hundreds of organisms studied [51]. Phosphorylation sites observed in the present study showed a higher degree of conservation when compared to human versus mouse, rat or cow ortholog proteins. The aforementioned study also detected a closer match between phosphorylation sites of general *canis lupus familiaris* and *homo sapiens* proteins compared to *mus musculus* and *rattus norvegicus* proteins. In some cases, as for SAA, phosphorylation was observed to occur in the extremes of α- helical motifs, suggesting that solvent-exposed residues could be more prone to phosphorylation by ecto-kinases. The action of ecto- kinases and ecto-phosphatases is still very poorly characterized but there are evidences of extensive cell surface and serum protein phosphorylation in cell culture medium [52] and in extracellular spaces in which adenosine triphosphate (ATP) is released in secretory vesicles [6].

Many of the observed phosphorylation sites show a high probability to be substrate of protein kinases CKI and CKII in agreement with recent findings suggesting a major role of atypical protein kinases that localize within the Golgi apparatus (Fam20C kinase family) that phosphorylates secretory pathway proteins within S-x-E motifs [53, 54] Our results suggest the involvement of other kinases that could play distinct roles in processes occurring in serum. Further investigation might reveal the intrincate kinase networks in extracellular space.

Quantitative data showed that α-2-HS-glycoprotein / Fetuin-A is the most abundant phosphoprotein in dog serum (with 50%-70% of all phosphopeptide intensities attributed to this protein), with kininogen-1 second in abundance (with 16–20% of intensity attributed to phosphopeptides). Previous studies have indicated fetuin-A to be an abundant phosphoprotein in human serum [31] based on spectral counts. Recently, phosphorylated fetuin-A has been observed to play an important role in insulin resistance [55] and procalcific milieus [56]. Its synthesis is divergently regulated in response to injury or infection, classifying it as a negative or positive acute phase protein (APP) [57]. Liver expression of fetuin-A is negatively regulated by several proinflammatory cytokines (such as TNF, IL-1, IL-6 and IFN-γ) and its concentration in systemic blood declines during inflammation in patients under dialysis, inversely correlating with the level of the acute phase protein (CRP) [58]. It also plays a role in augmentation of phagocytosis of neutrophils by macrophages, thus acting as an anti-inflammatory molecule [59].

Serum amyloid A (SAA), displays a very significant increase in phosphorylation at S 101 (p = 0.00018) in individual babesiosis samples and the highest increase in the TMT-labeled babesiosis pool both in phosphorylated and non-phosphorylated forms of all proteins observed. Some ELISA-based quantification studies have shown an 800-fold increase of SAA levels in canine babesiosis [32] and significant increases have been registered in canine monocytic ehrlichiosis [60]. On the other hand, studies based on immunoturbidimetric methods have found barely detectable levels of SAA and no significant variations with babesiosis [33] or leishmaniosis [61].

SAA has been reported to promote chemotaxis, migration, and adhesion of monocytes/macrophages [62]. Recent findings have detected differences between recombinant SAA and native SAA isoforms, suggesting an importance of post-translational modifications in the activity of this multi-functional protein [63], though the role of SAA phosphorylation is not yet understood. After monokine-induced hepatic secretion of SAA, it associates with high-density lipoprotein (HDL) in the circulation as apo-SAA. Plasma clearance of apo-SAA is more rapid than any of the other HDL apolipoproteins and preferentially associates with neutrophil membranes [64]. Our results revealed that another component of HDL, apolipoprotein AI (apoAI), is phosphorylated in dog serum at S211 and S220, supporting the hypothesis that HDL-apoprotein phosphorylation might play an important role in lipid clearance in babesiosis. In fact, human apoA1 phosphorylation has been observed at S201 during both synthesis and secretion from HepG2 and primary human hepatocytes [65]. Phosphorylation of HDL protein components such as apoAI and SAA by ecto- kinases (*i*.*e*., calcium/calmodulin-dependent serine protein kinases secreted by platelets [66]) may play a key role in HDL function. Our data together with recent findings [7] strongly suggest further investigation on phosphorylation status variations of HDL protein constituents.

It is known that, as in many infectious diseases affecting humans, canine babesiosis elicits the exacerbated secretion of acute phase proteins (APP) [32]. A recent proteomic analysis of dog serum comparing babesiosis-affected and healthy individuals showed changes in levels of proteins involved in acute phase response, complement factor and the coagulation cascade, as well as of lipoproteins [67]. When comparing acute phase protein levels previously reported for various infectious diseases [68], although some proteins, such as SAA and CRP, are generally upregulated in multiple infections, fold changes were found to be quite different depending on the type of infectious agent. In case of SAA, the most prominent upregulation is observed in malaria (caused by *Plasmodium Falciparum* and *Plasmodium vivax*) and in SARS [68]. Moreover, in dogs infected with *B*. *canis*, the reported diagnostic sensitivity of CRP, SAA and platelet counts is significantly higher than haptoglobin, erythrocyte sedimentation rate, hematocrit and white blood cell count [32].

We obserbed as well a significant level reduction in Kininogen 1 phosphorylated at T326 (p = 0.0257). Phosphorylated kininogen1 has been previously reported in human serum [31]. High molecular weight-kininogen (HMW-kininogen) plays an important role in blood coagulation by helping to position prekallikrein and factor XI next to factor XII. It also inhibits thrombin- and plasmin-induced aggregation of thrombocytes [69]. Soluble parasite antigens from *Babesia canis* have been reported to be insufficient to activate the kallikrein system in dogs infected with *Babesia canis* [70]. There is no evidence of the role of kininogen phosphorylation on the function of kininogen 1, but its involvement in platelet degranulation, its ability to bind platelet and its implication in disseminated intravascular coagulation [71] could explain the detected alteration in phosphokininogen 1.

Similarly, fibrinogen α /fibrinopeptide A phosphorylated at both T20 (p = 0.0257) and S22 (p = 0.0193) was found to be significantly down-phosphorylated in individual serum samples from babesiotic dogs compared to healthy controls. Another phosphoprotein is involved in coagulation, fibrinogen beta/ fibrinopeptide B (phosphorylated at T35), suggesting an influence of modifications on hemostasis. Phosphorylation has been previously observed as well in coagulation factor V, FVIII, FIX and prothrombin [72]. During the conversion of fibrinogen to fibrin, thrombin cleaves fibrinopeptide A and B located on the N-terminus of the fibrinogen α and β chains, respectively [73]. The resulting monomers polymerize end to end to progressively form protofibrils, fibrin fibers and fibrin gel. Increased phosphorylation of Fibrinopeptide A in acute phase [74] leads to an approximately 65% increase in substrate specificity towards hydrolysis of fibrinogen Aα (1–20) [75]. Other proteins involved in coagulation such as coagulation factor V were differentially phosphorylated, although not significantly in our data. Plasma-derived factor Va, the major secretory platelet phosphoprotein [76], is phosphorylated in the presence of thrombin stimulated platelets on both the heavy and the light chain [77].

Lower levels of phosphorylated coagulation factors observed in our data might be related to thrombocytopenia observed in *B*.*canis*-caused babesiosis [33, 78] as well as to the elevated serum phosphate level in non-survivor babesiotic dogs [33]. Moreover, alteration of phosphorylation levels of acute phase proteins (SAA as the clearest example in our data) and proteins involved in coagulation / platelet degranulation (fibrinogen α and kininogen 1) could indicate an increased adhesion of the phosphorylated form of these proteins to endothelial tissue or to erythrocyte debris generated by hemolysis, a common effect of babesiosis. Indeed, together with other alterations observed in our data (such as α-2- antiplasmin and other fibrinogens) these changes could reflect alterations in fibrinolysis previously observed in canine babesiosis [79].

In summary, our qualitative individual sample data showed novel and conserved phosphorylation sites in canine serum phosphoproteins and significant variations in a number of phosphoprotein levels upon babesiosis. In addition, quantitative data are valuable to establish relative abundances of serum phosphoproteins jointly offering an initial characterization of variations occurring in babesiosis. Further investigation with a larger number of samples and more extensive enrichment is required to increase the number of detected phosphoproteins. Further *in vivo* experiments are needed to elucidate the role of phosphorylation of many serum proteins as well as to characterize the role of ecto- kinases and ecto-phosphatases in modulating extracellular protein phosphorylation.

## Conclusions

The present analysis represents to the best of our knowledge the first characterization of canine serum phosphoproteins and the first approach to map modifications of serum protein phosphorylation status elicited by babesiosis. Phosphorylation sites detected in canine serum proteins showed a higher degree of conservation when compared with human than with other mammalian species present in phosphorylation databases. Additionally, the results show that the canine serum phosphoproteome is characterized by a wide dynamic range, with fetuin-A and kininogen-1 as major phosphoproteins. Moreover, significant differences in SAA, kininogen 1 and fibrinogen α phosphorylation levels were detected in canine babesiosis that could be useful as candidate biomarkers. These findings suggest that extracellular phosphorylation / de-phosphorylation catalyzed by extracellular kinases and phosphatases, a field which is gaining much attention in recent years, could be involved in relevant physiological processes related with infectious diseases.

## Supporting information

S1 Table Clinical values and significantly varying phosphopeptides. (XLSX)

S2 Table Phosphopeptides: MS parameters individual samples and TMT-labeled pools. (XLSX)

S1 Fig Phosphopeptides: MS spectra. (DOCX)

## Abbreviations

ApoA-I: apolipoprotein A-I
APP: acute phase protein
CRP: C-reactive protein
SAA: serum amyloid A
FASP: filter aided sample preparation
FVIII: coagulation factor 8
ITIH2: inter-alpha-trypsin inhibitor heavy chain H2
iTRAQ: isobaric tag for relative and absolute quantitation LC-MS/MS, Liquid chromatography-tandem mass spectrometry
PTM: post-translational modification
TMT: tandem mass tag
