# Supplementary material for: LC-MS/MS analysis of the dog serum phosphoproteome reveals novel and conserved phosphorylation sites: Phosphoprotein patterns in babesiosis caused by Babesia canis, a case study
Source: PLoS One. 2018 Nov 28;13(11):e0207245. doi: 10.1371/journal.pone.0207245 (PMC6261647; doi:10.1371/journal.pone.0207245)
Supplement: S1 Fig — (DOCX) [file pone.0207245.s003.docx]

**Fibrinopeptoide B**

**120106, 229215**

Sequence: HYYDDTDEEER, Y2-Phospho (79.96633 Da), Y3-Phospho (79.96633 Da)

Charge: +2, Monoisotopic m/z: 816.24585 Da (-4.53 mmu/-5.55 ppm), MH+: 1631.48442 Da, RT: 47.9800 min,

Identified with: Sequest HT (v1.17); XCorr:1.55, Percolator q-Value:0.00254, Percolator PEP:0.0561, ptmRS: Best Site Probabilities:Y2(Phospho): 100; Y3(Phospho): 100, Ions matched by search engine: 0/0

Fragment match tolerance used for search: 0.02 Da

Fragments used for search: b; b-H₂O; y; y-H₂O; y-NH₃

Protein references (2):

- RecName: Full=Fibrinogen beta chain; Contains: RecName: Full=Fibrinopeptide B; Contains: RecName: Full=Fibrinogen beta chain

- fibrinopeptide B

**Sulfhydryl oxidase**

**928139154**

Sequence: HNLDHSQETAEAQEVLQAIR, S6-Phospho (79.96633 Da), H1-TMT6plex (229.16293 Da)

Charge: +4, Monoisotopic m/z: 650.32178 Da (+3.56 mmu/+5.47 ppm), MH+: 2598.26528 Da, RT: 76.6666 min,

Identified with: Sequest HT (v1.17); XCorr:5.48, Percolator q-Value:0, Percolator PEP:0.000273, ptmRS: Best Site Probabilities:S6(Phospho): 100, Ions matched by search engine: 0/0

Fragment match tolerance used for search: 0.02 Da

Fragments used for search: b; b-H₂O; b-NH₃; y; y-H₂O; y-NH₃

Protein references (1):

- PREDICTED: LOW QUALITY PROTEIN: sulfhydryl oxidase 1, partial [Canis lupus familiaris]

**Chromogranin A**

**244539517**

Sequence: HPDSQAEEDSEGLSQGLVDTEK, S4-Phospho (79.96633 Da), K22-TMT6plex (229.16293 Da), H1-TMT6plex (229.16293 Da)

Charge: +3, Monoisotopic m/z: 970.45679 Da (+3.48 mmu/+3.59 ppm), MH+: 2909.35581 Da, RT: 73.4736 min,

Identified with: Sequest HT (v1.17); XCorr:2.47, Percolator q-Value:0.000698, Percolator PEP:0.0142, ptmRS: Best Site Probabilities:S4(Phospho): 100; K22(TMT6plex): 100, Ions matched by search engine: 0/0

Fragment match tolerance used for search: 0.02 Da

Fragments used for search: b; b-H₂O; b-NH₃; y; y-H₂O; y-NH₃

Protein references (2):

- chromogranin A, partial [Canis lupus familiaris]

- PREDICTED: chromogranin-A [Canis lupus familiaris]

**ITIH2**

**73949158**

Sequence: SVFGESGEVMEEADQVTLYSYK, S6-Phospho (79.96633 Da), K22-TMT6plex (229.16293 Da), M10-Oxidation (15.99492 Da), S1-TMT6plex (229.16293 Da)

Charge: +3, Monoisotopic m/z: 1008.14160 Da (+1.93 mmu/+1.91 ppm), MH+: 3022.41025 Da, RT: 102.5972 min,

Identified with: Sequest HT (v1.17); XCorr:2.74, Percolator q-Value:0, Percolator PEP:0.00368, ptmRS: Best Site Probabilities:S6(Phospho): 100; M10(Oxidation): 100; K22(TMT6plex): 100, Ions matched by search engine: 0/0

Fragment match tolerance used for search: 0.02 Da

Fragments used for search: b; b-H₂O; b-NH₃; y; y-H₂O; y-NH₃

Protein references (1):

- PREDICTED: inter-alpha-trypsin inhibitor heavy chain H2 [Canis lupus familiaris]

**Coagulation factor V**

**545504920**

Sequence: WHLVSEKGSYEIVPDAEDMAVDK, S5-Phospho (79.96633 Da), K7-TMT6plex (229.16293 Da), K23-TMT6plex (229.16293 Da), W1-TMT6plex (229.16293 Da)

Charge: +4, Monoisotopic m/z: 847.18378 Da (+3.47 mmu/+4.09 ppm), MH+: 3385.71328 Da, RT: 90.8196 min,

Identified with: Sequest HT (v1.17); XCorr:3.86, Percolator q-Value:0, Percolator PEP:0.00665, ptmRS: Best Site Probabilities:K7(TMT6plex): 100; S9(Phospho): 99.23; K23(TMT6plex): 100, Ions matched by search engine: 0/0

Fragment match tolerance used for search: 0.02 Da

Fragments used for search: b; b-H₂O; b-NH₃; y; y-H₂O; y-NH₃

Protein references (1):

- PREDICTED: coagulation factor V [Canis lupus familiaris]

|  |  |  |  | **928125053** | **PREDICTED: Golgi membrane protein 1 [Canis lupus familiaris]** |  |  |  |  |  |  |  |  |  |  |  | **Phospho [S276(99.4)]** |  |  |  |  |  |  |  |  |
| --- | --- | --- | --- | --- | --- | --- | --- | --- | --- | --- | --- | --- | --- | --- | --- | --- | --- | --- | --- | --- | --- | --- | --- | --- | --- |

Sequence: GETNEIQVTSEEEPQR, T9-Phospho (79.96633 Da), G1-TMT6plex (229.16293 Da)

Charge: +3, Monoisotopic m/z: 719.00220 Da (+5.57 mmu/+7.74 ppm), MH+: 2154.99204 Da, RT: 61.7026 min,

Identified with: Sequest HT (v1.17); XCorr:2.63, Percolator q-Value:0.000698, Percolator PEP:0.0336, ptmRS: Best Site Probabilities:S10(Phospho): 99.42, Ions matched by search engine: 0/0

Fragment match tolerance used for search: 0.02 Da

Fragments used for search: b; b-H₂O; b-NH₃; y; y-H₂O; y-NH₃

Protein references (1):

- PREDICTED: Golgi membrane protein 1 [Canis lupus familiaris]

**NOT TMT LABLELED**

**P0CG06**

- Ig lambda-3 chain C regions

sequence: SHKSYSCQVTHEGSTVEK, Y5-Phospho (79.96633 Da), C7-Carbamidomethyl (57.02146 Da)

Charge: +3, Monoisotopic m/z: 715.30774 Da (-0.92 mmu/-1.29 ppm), MH+: 2143.90866 Da, RT: 42.3684 min,

Identified with: Sequest HT (v1.17); XCorr:3.72, Percolator q-Value:0, Percolator PEP:1.35e-09, ptmRS: Best Site Probabilities:S4(Phospho): 49.86; S6(Phospho): 49.86, Ions matched by search engine: 0/0

Fragment match tolerance used for search: 0.02 Da

Fragments used for search: b; b-H₂O; b-NH₃; y; y-H₂O; y-NH₃

Protein references (1):

- Ig lambda-3 chain C regions

**P05093**

- Steroid 17-alpha-hydroxylase/17,20 lyase

Sequence: KKLYEEIDQNVGFSR, Y4-Phospho (79.96633 Da)

Charge: +5, Monoisotopic m/z: 381.98792 Da (-0.03 mmu/-0.08 ppm), MH+: 1905.91047 Da, RT: 23.9839 min,

Identified with: Sequest HT (v1.17); XCorr:1.19, Percolator q-Value:0.00186, Percolator PEP:0.0674, ptmRS: Best Site Probabilities:Y4(Phospho): 99.98, Ions matched by search engine: 0/0

Fragment match tolerance used for search: 0.02 Da

Fragments used for search: b; b-H₂O; b-NH₃; y; y-H₂O; y-NH₃

Protein references (1):

- Steroid 17-alpha-hydroxylase/17,20 lyase

**928124878**

- PREDICTED: WD repeat-containing protein 87 [Canis lupus familiaris]

Sequence: KQAQQLSSAMKEIPHLYPIR, S7-Phospho (79.96633 Da), S8-Phospho (79.96633 Da), Y17-Phospho (79.96633 Da)

Charge: +4, Monoisotopic m/z: 645.29504 Da (-2.66 mmu/-4.13 ppm), MH+: 2578.15835 Da, RT: 31.7896 min,

Identified with: Sequest HT (v1.17); XCorr:1.46, Percolator q-Value:0.00141, Percolator PEP:0.0531, ptmRS: Best Site Probabilities:S7(Phospho): 100; S8(Phospho): 100; Y17(Phospho): 100, Ions matched by search engine: 0/0

Fragment match tolerance used for search: 0.02 Da

Fragments used for search: b; b-H₂O; b-NH₃; y; y-H₂O; y-NH₃

Protein references (1):

- PREDICTED: WD repeat-containing protein 87 [Canis lupus familiaris]

**Q12923-4**

- Isoform 3 of Tyrosine-protein phosphatase non-receptor type 13

Sequence: KTTQVKDYSFVTEENTFEVK, T2-Phospho (79.96633 Da), T3-Phospho (79.96633 Da)

Charge: +5, Monoisotopic m/z: 511.42950 Da (-0.27 mmu/-0.53 ppm), MH+: 2553.11842 Da, RT: 38.3746 min,

Identified with: Sequest HT (v1.17); XCorr:1.31, Percolator q-Value:0.00186, Percolator PEP:0.0709, ptmRS: Best Site Probabilities:T2(Phospho): 99.94; T3(Phospho): 97.92, Ions matched by search engine: 0/0

Fragment match tolerance used for search: 0.02 Da

Fragments used for search: b; b-H₂O; b-NH₃; y; y-H₂O; y-NH₃

Protein references (4):

- Isoform 2 of Tyrosine-protein phosphatase non-receptor type 13

- Isoform 4 of Tyrosine-protein phosphatase non-receptor type 13

- Tyrosine-protein phosphatase non-receptor type 13

- Isoform 3 of Tyrosine-protein phosphatase non-receptor type 13

**Q13255**

- Metabotropic glutamate receptor 1

Sequence: KAGAGNANSNGKSVSWSEPGGGQVPK, S13-Phospho (79.96633 Da), S15-Phospho (79.96633 Da)

Charge: +5, Monoisotopic m/z: 529.63660 Da (-0.28 mmu/-0.52 ppm), MH+: 2644.15388 Da, RT: 36.9626 min,

Identified with: Sequest HT (v1.17); XCorr:1.55, Percolator q-Value:0.00186, Percolator PEP:0.0701, ptmRS: Best Site Probabilities:S13(Phospho): 66.05; S15(Phospho): 66.05; S17(Phospho): 66.05, Ions matched by search engine: 0/0

Fragment match tolerance used for search: 0.02 Da

Fragments used for search: b; b-H₂O; b-NH₃; y; y-H₂O; y-NH₃

Protein references (1):

- Metabotropic glutamate receptor 1

**Q13255**

Sequence: KAGAGNANSNGKSVSWSEPGGGQVPK, S15-Phospho (79.96633 Da), S17-Phospho (79.96633 Da)

Charge: +5, Monoisotopic m/z: 529.63660 Da (-0.28 mmu/-0.52 ppm), MH+: 2644.15388 Da, RT: 36.9626 min,

Identified with: Sequest HT (v1.17); XCorr:1.53, Percolator q-Value:0.000728, Percolator PEP:0.0438, ptmRS: Best Site Probabilities:S13(Phospho): 66.05; S15(Phospho): 66.05; S17(Phospho): 66.05, Ions matched by search engine: 0/0

Fragment match tolerance used for search: 0.02 Da

Fragments used for search: b; b-H₂O; b-NH₃; y; y-H₂O; y-NH₃

Protein references (1):

- Metabotropic glutamate receptor 1

**Q3B726 (THERE ARE 6 phosphopeptides)**

- DNA-directed RNA polymerase I subunit RPA43

 equence: KKHQEVQDQDPVFQGSDSSGYQSDHK, S16-Phospho (79.96633 Da), S18-Phospho (79.96633 Da)

Charge: +4, Monoisotopic m/z: 784.32867 Da (+1.01 mmu/+1.28 ppm), MH+: 3134.29287 Da, RT: 38.3043 min,

Identified with: Sequest HT (v1.17); XCorr:0.79, Percolator q-Value:0.00148, Percolator PEP:0.0822, ptmRS: Best Site Probabilities:S16(Phospho): 50.21; S18(Phospho): 50.21; S19(Phospho): 97.77, Ions matched by search engine: 0/0

Fragment match tolerance used for search: 0.02 Da

Fragments used for search: b; b-H₂O; b-NH₃; y; y-H₂O; y-NH₃

Protein references (1):

- DNA-directed RNA polymerase I subunit RPA43

**Q3B726**

- DNA-directed RNA polymerase I subunit RPA43

Sequence: KKHQEVQDQDPVFQGSDSSGYQSDHK, S16-Phospho (79.96633 Da), S18-Phospho (79.96633 Da)

Charge: +4, Monoisotopic m/z: 784.32867 Da (+1.01 mmu/+1.28 ppm), MH+: 3134.29287 Da, RT: 38.3043 min,

Identified with: Sequest HT (v1.17); XCorr:0.79, Percolator q-Value:0.00148, Percolator PEP:0.0822, ptmRS: Best Site Probabilities:S16(Phospho): 50.21; S18(Phospho): 50.21; S19(Phospho): 97.77, Ions matched by search engine: 0/0

Fragment match tolerance used for search: 0.02 Da

Fragments used for search: b; b-H₂O; b-NH₃; y; y-H₂O; y-NH₃

Protein references (1):

- DNA-directed RNA polymerase I subunit RPA43

Sequence: KKHQEVQDQDPVFQGSDSSGYQSDHK, S18-Phospho (79.96633 Da), S19-Phospho (79.96633 Da)

Charge: +4, Monoisotopic m/z: 784.32867 Da (+1.01 mmu/+1.28 ppm), MH+: 3134.29287 Da, RT: 38.3043 min,

Identified with: Sequest HT (v1.17); XCorr:0.79, Percolator q-Value:0.00148, Percolator PEP:0.0825, ptmRS: Best Site Probabilities:S16(Phospho): 50.21; S18(Phospho): 50.21; S19(Phospho): 97.77, Ions matched by search engine: 0/0

Fragment match tolerance used for search: 0.02 Da

Fragments used for search: b; b-H₂O; b-NH₃; y; y-H₂O; y-NH₃

Protein references (1):

- DNA-directed RNA polymerase I subunit RPA43

Sequence: KKHQEVQDQDPVFQGSDSSGYQSDHK, Y21-Phospho (79.96633 Da), S23-Phospho (79.96633 Da)

Charge: +3, Monoisotopic m/z: 1045.43555 Da (+1.08 mmu/+1.04 ppm), MH+: 3134.29209 Da, RT: 42.6161 min,

Identified with: Sequest HT (v1.17); XCorr:0.48, Percolator q-Value:0.00774, Percolator PEP:0.207, ptmRS: Best Site Probabilities:S16(Phospho): 40; S18(Phospho): 40; S19(Phospho): 40; Y21(Phospho): 40; S23(Phospho): 40, Ions matched by search engine: 0/0

Fragment match tolerance used for search: 0.02 Da

Fragments used for search: b; b-H₂O; b-NH₃; y; y-H₂O; y-NH₃

Protein references (1):

- DNA-directed RNA polymerase I subunit RPA43

Sequence: KKHQEVQDQDPVFQGSDSSGYQSDHK, S19-Phospho (79.96633 Da), S23-Phospho (79.96633 Da)

Charge: +3, Monoisotopic m/z: 1045.43555 Da (+1.08 mmu/+1.04 ppm), MH+: 3134.29209 Da, RT: 42.6161 min,

Identified with: Sequest HT (v1.17); XCorr:0.48, Percolator q-Value:0.00774, Percolator PEP:0.207, ptmRS: Best Site Probabilities:S16(Phospho): 40; S18(Phospho): 40; S19(Phospho): 40; Y21(Phospho): 40; S23(Phospho): 40, Ions matched by search engine: 0/0

Fragment match tolerance used for search: 0.02 Da

Fragments used for search: b; b-H₂O; b-NH₃; y; y-H₂O; y-NH₃

Protein references (1):

- DNA-directed RNA polymerase I subunit RPA43

**P24593**

**- PREDICTED: insulin-like growth factor-binding protein 5 [Canis lupus familiaris]**

Sequence: IERDSREHEEPTTSEMAEETYSPK, S5-Phospho (79.96633 Da)

Charge: +4, Monoisotopic m/z: 733.56482 Da (+0.61 mmu/+0.83 ppm), MH+: 2931.23745 Da, RT: 41.8195 min,

Identified with: Sequest HT (v1.17); XCorr:1.03, Percolator q-Value:0.00455, Percolator PEP:0.172, ptmRS: Best Site Probabilities:S5(Phospho): 97.07, Ions matched by search engine: 0/0

Fragment match tolerance used for search: 0.02 Da

Fragments used for search: b; b-H₂O; b-NH₃; y; y-H₂O; y-NH₃

Protein references (2):

- Insulin-like growth factor-binding protein 5

- PREDICTED: insulin-like growth factor-binding protein 5 [Canis lupus familiaris]

**74005944**

Complement factor H

Sequence: SSIFSEEIEETSKPK, S5-Phospho (79.96633 Da)

Charge: +3, Monoisotopic m/z: 597.60840 Da (+0.36 mmu/+0.6 ppm), MH+: 1790.81064 Da, RT: 64.0763 min,

Identified with: Sequest HT (v1.17); XCorr:1.55, Percolator q-Value:0.00132, Percolator PEP:0.052, ptmRS: Best Site Probabilities:S5(Phospho): 99.98, Ions matched by search engine: 0/0

Fragment match tolerance used for search: 0.02 Da

Fragments used for search: b; b-H₂O; b-NH₃; y; y-H₂O; y-NH₃

Protein references (1):

- PREDICTED: complement factor H [Canis lupus familiaris]

**928157557**

**Kanadaptin**

Sequence: LQQETELEEAVQDTRPPTDLMCSKETK, T5-Phospho (79.96633 Da), T14-Phospho (79.96633 Da), C22-Carbamidomethyl (57.02146 Da), M21-Oxidation (15.99492 Da)

Charge: +5, Monoisotopic m/z: 671.29803 Da (+4.99 mmu/+7.43 ppm), MH+: 3352.46107 Da, RT: 39.4128 min,

Identified with: Sequest HT (v1.17); XCorr:2.63, Percolator q-Value:0.00102, Percolator PEP:0.0813, ptmRS: Best Site Probabilities:T5(Phospho): 100; T14(Phospho): 100; M21(Oxidation): 100, Ions matched by search engine: 0/0

Fragment match tolerance used for search: 0.02 Da

Fragments used for search: b; b-H₂O; b-NH₃; y; y-H₂O; y-NH₃

Protein references (3):

- PREDICTED: kanadaptin isoform X3 [Canis lupus familiaris]

- PREDICTED: kanadaptin isoform X2 [Canis lupus familiaris]

- PREDICTED: kanadaptin isoform X1 [Canis lupus familiaris]

**545536330**

beta-arrestin-1

Sequence: GVKDDKEEEEDGTGSPQLNDR, S15-Phospho (79.96633 Da)

Charge: +3, Monoisotopic m/z: 800.00592 Da (-0.33 mmu/-0.41 ppm), MH+: 2398.00321 Da, RT: 30.0600 min,

Identified with: Sequest HT (v1.17); XCorr: 1.07, Percolator q-Value:0.00102, Percolator PEP:0.0691, ptmRS: Best Site Probabilities:S15(Phospho): 100, Ions matched by search engine: 0/0

Fragment match tolerance used for search: 0.02 Da

Fragments used for search: b; b-H₂O; b-NH₃; y; y-H₂O; y-NH₃

Protein references (4):

- PREDICTED: beta-arrestin-1 isoform X5 [Canis lupus familiaris]

- PREDICTED: beta-arrestin-1 isoform X1 [Canis lupus familiaris]

- PREDICTED: beta-arrestin-1 isoform X2 [Canis lupus familiaris]

- PREDICTED: beta-arrestin-1 isoform X4 [Canis lupus familiaris]

**359321488**

insulin-like growth factor-binding protein 3

****Sequence: YKVDYESQSTDTQNFSSEYKR, S9-Phospho (79.96633 Da), S16-Phospho (79.96633 Da)

Charge: +3, Monoisotopic m/z: 912.36884 Da (+0.33 mmu/+0.36 ppm), MH+: 2735.09195 Da, RT: 51.7532 min,

Identified with: Sequest HT (v1.17); XCorr:1.69, Percolator q-Value:0, Percolator PEP:0.000769, ptmRS: Best Site Probabilities:S9(Phospho): 33.29; T10(Phospho): 33.29; T12(Phospho): 33.29; S16(Phospho): 99.62, Ions matched by search engine: 0/0

Fragment match tolerance used for search: 0.02 Da

Fragments used for search: b; b-H₂O; b-NH₃; y; y-H₂O; y-NH₃

Protein references (1):

- PREDICTED: insulin-like growth factor-binding protein 3 [Canis lupus familiaris]
